# μ-Hydroxido-bis­[(2,2′-bipyridine)tricarbonyl­rhenium(I)] perrhenate

**DOI:** 10.1107/S160053680800490X

**Published:** 2008-02-27

**Authors:** Kai Ruth, Thorsten Morawitz, Hans-Wolfram Lerner, Michael Bolte

**Affiliations:** a Institut für Anorganische Chemie, J. W. Goethe-Universität Frankfurt, Max-von-Laue-Strasse 7, 60438 Frankfurt/Main, Germany

## Abstract

The title compound, [Re_2_(OH)(C_10_H_8_N_2_)_2_(CO)_6_][ReO_4_], is a mixed-valence rhenium compound containing discrete anions and cations. The Re^I^ atoms are in a slightly distorted octa­hedral environment, whereas the Re^VII^ atoms show the typical tetra­hedral coordination mode. The dihedral angle between the two bipyridine groups is 34.3 (7)°.

## Related literature

For related literature, see: Gibson *et al.* (2003 ▶).
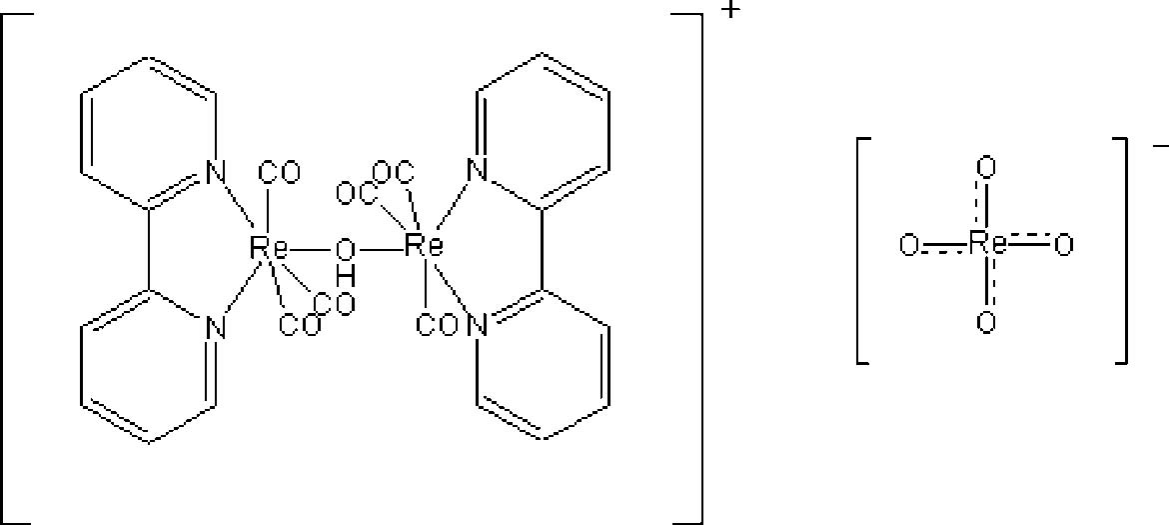


## Experimental

### 

#### Crystal data

[Re_2_(OH)(C_10_H_8_N_2_)_2_(CO)_6_][ReO_4_]
*M* *_r_* = 1120.04
Triclinic, 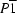

*a* = 9.0304 (7) Å
*b* = 11.0506 (9) Å
*c* = 15.5152 (13) Å
α = 96.488 (7)°
β = 94.768 (7)°
γ = 104.038 (6)°
*V* = 1482.6 (2) Å^3^
*Z* = 2
Mo *K*α radiation
μ = 12.28 mm^−1^
*T* = 173 (2) K
0.16 × 0.15 × 0.14 mm

#### Data collection

Stoe IPDSII two-circle diffractometer
Absorption correction: multi-scan (*MULABS*; Spek, 2003 ▶; Blessing, 1995 ▶) *T*_min_ = 0.142, *T*_max_ = 0.188
21159 measured reflections
5550 independent reflections
4782 reflections with *I* > 2σ(*I*)
*R*_int_ = 0.090

#### Refinement

*R*[*F* ^2^ > 2σ(*F* ^2^)] = 0.092
*wR*(*F* ^2^) = 0.262
*S* = 1.05
5550 reflections
398 parameters
H-atom parameters constrained
Δρ_max_ = 4.57 e Å^−3^
Δρ_min_ = −5.65 e Å^−3^

### 

Data collection: *X-AREA* (Stoe & Cie, 2001 ▶); cell refinement: *X-AREA*; data reduction: *X-AREA*; program(s) used to solve structure: *SHELXS97* (Sheldrick, 2008 ▶); program(s) used to refine structure: *SHELXL97* (Sheldrick, 2008 ▶); molecular graphics: *XP* in *SHELXTL-Plus* (Sheldrick, 2008 ▶); software used to prepare material for publication: *SHELXL97*.

## Supplementary Material

Crystal structure: contains datablocks I, global. DOI: 10.1107/S160053680800490X/at2546sup1.cif

Structure factors: contains datablocks I. DOI: 10.1107/S160053680800490X/at2546Isup2.hkl

Additional supplementary materials:  crystallographic information; 3D view; checkCIF report

## Figures and Tables

**Fig. 1.**
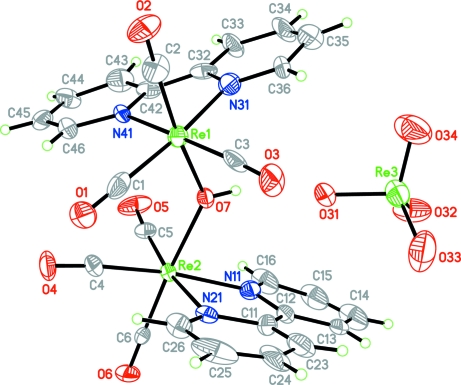
Perspective view of the title compound with the atom numbering scheme; displacement ellipsoids are at the 30% probability level. H atoms are drawn as small spheres of arbitrary radii.

## supplementary crystallographic information

### Comment

We report here the X-ray crystal structure analysis of the mixed-valence rhenium compound [(Re(CO)_3_bipy)_2_OH]^+^[ReO_4_]^-^ (bipy = C_10_H_6_N_2_). Thereby Re features in the cation an oxidation state +1 whereas in the perrhenate anion the Re center possesses the oxidation number +7. Surprisingly we have obtained the title compound as an oxidation and hydrolysis product of [Re(CO)_3_(bipy)O_3_SCF_3_]. X-ray quality crystals of the title compound were grown by diffusion of hexane into a tetrahydrofuran solution of the mixed-valence rhenium compound [(Re(CO)_3_bipy)_2_OH]^+^[ReO_4_]^-^ at ambient temperature.

The title compound, [C_26_H_17_N_4_O_7_Re_2_]^+^[ReO_4_]^-^, is a mixed-valence rhenium compound containing discrete anions and cations. The Re^I^ atoms are in a slightly distorted octahedral environment, whereas the Re^VII^ atoms show the typical tetrahedral coordination mode. The dihedral angle between the two bipyridine moieties is 34.3 (7)°. A comparable compound with the bipyridine residues substituted by methyl groups in the *para*-position to the N atoms was determined by Gibson *et al.* (2003).

### Experimental

The title compound was obtained as an oxidation and hydrolysis product of [Re(CO)_3_(bipy)O_3_SCF_3_] from a mixture of [Re(CO)_3_(bipy)O_3_SCF_3_] (54 mg, 0.09 mmol) and 17.5 ml tetrahydrofuran. X-ray quality crystals of the title compound were grown by diffusion of hexane into a solution of the mixed-valence rhenium compound [(Re(CO)_3_bipy)_2_OH]^+^[ReO_4_]^-^ in tetrahydrofuran at ambient temperature.

### Refinement

H atoms were geometrically positioned and refined using a riding model with fixed individual displacement parameters [U(H) = 1.2 *U*_eq_(C,*O*)] and with O—H = 0.82Å and C—H = 0.95 Å. The highest peak (4.59 e.Å^-3^) in the final difference electron density map is at 0.60Å from Re1 and the deepest hole (-5.66 e.Å^-3^) is at 0.33Å from Re3.

### Crystal data

**Table:**

[Re_2_(OH)(C_10_H_8_N_2_)_2_(CO)_6_][ReO_4_]	*Z* = 2
*M**_r_* = 1120.04	*F*_000_ = 1028
Triclinic, *P*1	*D*_x_ = 2.509 Mg m^−^^3^
Hall symbol: -P 1	Mo *K*α radiation λ = 0.71073 Å
*a* = 9.0304 (7) Å	Cell parameters from 19460 reflections
*b* = 11.0506 (9) Å	θ = 3.8–25.6º
*c* = 15.5152 (13) Å	µ = 12.28 mm^−^^1^
α = 96.488 (7)º	*T* = 173 (2) K
β = 94.768 (7)º	Block, orange
γ = 104.038 (6)º	0.16 × 0.15 × 0.14 mm
*V* = 1482.6 (2) Å^3^	

### Data collection

**Table:**

Stoe IPDSII two-circle diffractometer	5550 independent reflections
Radiation source: fine-focus sealed tube	4782 reflections with *I* > 2σ(*I*)
Monochromator: graphite	*R*_int_ = 0.090
*T* = 173(2) K	θ_max_ = 25.7º
ω scans	θ_min_ = 3.7º
Absorption correction: multi-scan(MULABS; Spek, 2003; Blessing, 1995)	*h* = −10→10
*T*_min_ = 0.143, *T*_max_ = 0.188	*k* = −13→13
21159 measured reflections	*l* = −18→18

### Refinement

**Table:**

Refinement on *F*^2^	Hydrogen site location: inferred from neighbouring sites
Least-squares matrix: full	H-atom parameters constrained
*R*[*F*^2^ > 2σ(*F*^2^)] = 0.092	*w* = 1/[σ^2^(*F*_o_^2^) + (0.1565*P*)^2^ + 50.8834*P*] where *P* = (*F*_o_^2^ + 2*F*_c_^2^)/3
*wR*(*F*^2^) = 0.262	(Δ/σ)_max_ < 0.001
*S* = 1.05	Δρ_max_ = 4.57 e Å^−^^3^
5550 reflections	Δρ_min_ = −5.65 e Å^−^^3^
398 parameters	Extinction correction: SHELXL97 (Sheldrick, 2008), Fc^*^=kFc[1+0.001xFc^2^λ^3^/sin(2θ)]^-1/4^
Primary atom site location: structure-invariant direct methods	Extinction coefficient: 0.0058 (8)
Secondary atom site location: difference Fourier map	

### Special details

**Table:**

Geometry. All e.s.d.'s (except the e.s.d. in the dihedral angle between two l.s. planes) are estimated using the full covariance matrix. The cell e.s.d.'s are taken into account individually in the estimation of e.s.d.'s in distances, angles and torsion angles; correlations between e.s.d.'s in cell parameters are only used when they are defined by crystal symmetry. An approximate (isotropic) treatment of cell e.s.d.'s is used for estimating e.s.d.'s involving l.s. planes.
Refinement. Refinement of *F*^2^ against ALL reflections. The weighted *R*-factor *wR* and goodness of fit *S* are based on *F*^2^, conventional *R*-factors *R* are based on *F*, with *F* set to zero for negative *F*^2^. The threshold expression of *F*^2^ > σ(*F*^2^) is used only for calculating *R*-factors(gt) *etc*. and is not relevant to the choice of reflections for refinement. *R*-factors based on *F*^2^ are statistically about twice as large as those based on *F*, and *R*- factors based on ALL data will be even larger.

### Fractional atomic coordinates and isotropic or equivalent isotropic displacement parameters (Å^2^)

**Table:**

	*x*	*y*	*z*	*U*_iso_*/*U*_eq_	
Re1	0.76687 (9)	0.89450 (8)	0.70601 (5)	0.0392 (3)	
C1	0.770 (3)	1.024 (3)	0.806 (2)	0.057 (7)	
O1	0.771 (3)	1.0983 (16)	0.8628 (12)	0.069 (5)	
C2	0.910 (3)	1.022 (3)	0.657 (2)	0.062 (7)	
O2	1.004 (2)	1.0976 (19)	0.6342 (15)	0.073 (6)	
C3	0.933 (3)	0.858 (2)	0.7703 (14)	0.052 (6)	
O3	1.037 (2)	0.839 (2)	0.8152 (13)	0.071 (5)	
Re2	0.42562 (9)	0.72867 (7)	0.83203 (5)	0.0352 (3)	
C4	0.419 (3)	0.901 (2)	0.8481 (17)	0.052 (6)	
O4	0.413 (3)	1.0039 (17)	0.8637 (16)	0.077 (6)	
C5	0.270 (3)	0.706 (2)	0.7326 (16)	0.052 (6)	
O5	0.181 (2)	0.689 (3)	0.6702 (13)	0.081 (7)	
C6	0.266 (3)	0.705 (2)	0.9084 (13)	0.044 (5)	
O6	0.175 (2)	0.6920 (19)	0.9559 (11)	0.061 (5)	
O7	0.6037 (16)	0.7393 (13)	0.7439 (9)	0.038 (3)	
H7	0.6092	0.6704	0.7208	0.046*	
N11	0.441 (2)	0.5359 (17)	0.8331 (12)	0.044 (4)	
C11	0.664 (3)	0.626 (2)	0.9353 (15)	0.046 (5)	
C12	0.554 (4)	0.515 (2)	0.8855 (18)	0.062 (8)	
C13	0.574 (5)	0.394 (3)	0.891 (2)	0.093 (14)	
H13	0.6594	0.3841	0.9270	0.111*	
C14	0.475 (3)	0.290 (3)	0.845 (2)	0.093 (6)	
H14	0.4887	0.2082	0.8462	0.112*	
C15	0.349 (4)	0.313 (2)	0.796 (2)	0.093 (6)	
H15	0.2705	0.2424	0.7682	0.112*	
C16	0.333 (5)	0.4335 (19)	0.786 (2)	0.093 (6)	
H16	0.2511	0.4449	0.7476	0.111*	
N21	0.620 (2)	0.7420 (17)	0.9301 (11)	0.040 (4)	
C23	0.801 (4)	0.630 (4)	0.9854 (18)	0.083 (11)	
H23	0.8352	0.5555	0.9873	0.100*	
C24	0.883 (4)	0.739 (4)	1.031 (2)	0.079 (10)	
H24	0.9750	0.7386	1.0654	0.095*	
C25	0.846 (3)	0.850 (5)	1.0319 (17)	0.094 (14)	
H25	0.9072	0.9244	1.0663	0.113*	
C26	0.701 (3)	0.849 (2)	0.9750 (16)	0.053 (6)	
H26	0.6697	0.9247	0.9717	0.063*	
N31	0.728 (2)	0.755 (2)	0.5863 (14)	0.051 (5)	
C32	0.602 (3)	0.747 (2)	0.5346 (13)	0.050 (6)	
C33	0.560 (4)	0.651 (3)	0.4601 (17)	0.064 (7)	
H33	0.4649	0.6393	0.4249	0.077*	
C34	0.656 (4)	0.577 (4)	0.4399 (18)	0.087 (12)	
H34	0.6272	0.5122	0.3915	0.104*	
C35	0.795 (5)	0.597 (3)	0.490 (2)	0.083 (9)	
H35	0.8670	0.5513	0.4741	0.100*	
C36	0.828 (4)	0.687 (3)	0.5675 (17)	0.059 (7)	
H36	0.9202	0.6991	0.6050	0.071*	
N41	0.565 (2)	0.9191 (17)	0.6320 (11)	0.040 (4)	
C42	0.501 (2)	0.826 (2)	0.5597 (13)	0.043 (5)	
C43	0.365 (4)	0.827 (3)	0.513 (2)	0.071 (8)	
H43	0.3297	0.7712	0.4605	0.085*	
C44	0.277 (3)	0.911 (3)	0.5419 (16)	0.057 (6)	
H44	0.1766	0.9049	0.5152	0.068*	
C45	0.349 (3)	1.003 (3)	0.6129 (17)	0.056 (6)	
H45	0.2990	1.0663	0.6312	0.067*	
C46	0.484 (3)	1.005 (2)	0.6555 (16)	0.047 (5)	
H46	0.5257	1.0682	0.7039	0.056*	
Re3	0.84808 (14)	0.42214 (11)	0.72586 (9)	0.0663 (4)	
O31	0.757 (3)	0.5413 (19)	0.7321 (13)	0.070 (5)	
O32	0.705 (3)	0.286 (2)	0.7035 (13)	0.092 (8)	
O33	0.930 (4)	0.422 (3)	0.831 (2)	0.126 (12)	
O34	0.922 (6)	0.429 (3)	0.639 (2)	0.157 (17)	

### Atomic displacement parameters (Å^2^)

**Table:**

	*U*^11^	*U*^22^	*U*^33^	*U*^12^	*U*^13^	*U*^23^
Re1	0.0339 (5)	0.0455 (5)	0.0412 (5)	0.0145 (3)	0.0033 (3)	0.0087 (3)
C1	0.042 (12)	0.061 (15)	0.080 (19)	0.022 (11)	0.010 (11)	0.038 (14)
O1	0.117 (17)	0.035 (8)	0.046 (10)	0.010 (9)	0.009 (10)	−0.014 (7)
C2	0.037 (12)	0.060 (15)	0.09 (2)	0.005 (11)	0.005 (12)	0.017 (14)
O2	0.063 (12)	0.063 (11)	0.087 (14)	−0.007 (9)	0.009 (10)	0.035 (10)
C3	0.062 (14)	0.065 (15)	0.029 (10)	0.026 (12)	0.012 (10)	−0.014 (10)
O3	0.057 (11)	0.098 (15)	0.066 (12)	0.047 (11)	−0.013 (9)	0.005 (10)
Re2	0.0400 (5)	0.0361 (5)	0.0358 (5)	0.0188 (3)	0.0109 (3)	0.0060 (3)
C4	0.057 (14)	0.055 (14)	0.057 (14)	0.031 (11)	0.027 (11)	0.012 (11)
O4	0.091 (14)	0.045 (10)	0.116 (18)	0.041 (10)	0.056 (13)	0.023 (10)
C5	0.061 (14)	0.055 (13)	0.050 (13)	0.018 (11)	0.031 (12)	0.025 (11)
O5	0.052 (10)	0.14 (2)	0.051 (11)	0.011 (11)	−0.008 (9)	0.047 (12)
C6	0.059 (13)	0.054 (12)	0.034 (10)	0.039 (11)	0.007 (9)	0.017 (9)
O6	0.069 (11)	0.080 (12)	0.048 (10)	0.027 (9)	0.032 (9)	0.025 (9)
O7	0.042 (7)	0.032 (7)	0.044 (8)	0.014 (6)	0.012 (6)	0.005 (6)
N11	0.047 (10)	0.041 (9)	0.047 (10)	0.008 (8)	0.016 (8)	0.012 (8)
C11	0.043 (11)	0.061 (13)	0.045 (12)	0.027 (10)	0.009 (9)	0.015 (10)
C12	0.10 (2)	0.050 (13)	0.058 (15)	0.045 (14)	0.051 (15)	0.025 (12)
C13	0.18 (4)	0.09 (2)	0.066 (18)	0.10 (3)	0.08 (2)	0.053 (17)
C14	0.144 (16)	0.030 (7)	0.103 (14)	0.003 (9)	0.071 (12)	−0.001 (8)
C15	0.144 (16)	0.030 (7)	0.103 (14)	0.003 (9)	0.071 (12)	−0.001 (8)
C16	0.144 (16)	0.030 (7)	0.103 (14)	0.003 (9)	0.071 (12)	−0.001 (8)
N21	0.041 (9)	0.045 (9)	0.031 (8)	0.008 (7)	0.013 (7)	0.003 (7)
C23	0.08 (2)	0.15 (3)	0.041 (14)	0.07 (2)	0.011 (14)	0.004 (18)
C24	0.059 (17)	0.13 (3)	0.065 (19)	0.05 (2)	0.036 (15)	0.01 (2)
C25	0.041 (14)	0.18 (4)	0.034 (13)	−0.006 (19)	−0.008 (11)	−0.025 (18)
C26	0.051 (13)	0.045 (12)	0.052 (13)	−0.002 (10)	0.006 (10)	−0.008 (10)
N31	0.042 (10)	0.058 (12)	0.058 (12)	0.016 (9)	0.011 (9)	0.018 (9)
C32	0.060 (14)	0.062 (14)	0.019 (9)	0.000 (11)	0.000 (9)	0.007 (9)
C33	0.081 (18)	0.067 (16)	0.046 (14)	0.032 (14)	0.013 (12)	−0.017 (12)
C34	0.12 (3)	0.12 (3)	0.037 (14)	0.08 (3)	0.024 (17)	−0.007 (16)
C35	0.10 (3)	0.08 (2)	0.08 (2)	0.035 (19)	0.04 (2)	0.016 (17)
C36	0.086 (18)	0.062 (15)	0.051 (14)	0.051 (14)	0.025 (13)	0.014 (11)
N41	0.046 (9)	0.049 (10)	0.036 (9)	0.029 (8)	0.015 (7)	0.010 (7)
C42	0.042 (11)	0.054 (12)	0.026 (9)	0.000 (9)	0.001 (8)	0.002 (9)
C43	0.064 (17)	0.08 (2)	0.067 (18)	0.011 (15)	0.010 (14)	0.017 (15)
C44	0.033 (11)	0.10 (2)	0.043 (12)	0.029 (12)	0.005 (9)	0.012 (13)
C45	0.064 (15)	0.069 (16)	0.052 (14)	0.036 (13)	0.014 (11)	0.030 (12)
C46	0.048 (12)	0.050 (12)	0.049 (13)	0.022 (10)	0.014 (10)	0.008 (10)
Re3	0.0648 (7)	0.0588 (7)	0.0775 (9)	0.0190 (5)	0.0181 (6)	0.0039 (6)
O31	0.095 (14)	0.065 (11)	0.068 (12)	0.045 (11)	0.037 (11)	0.016 (9)
O32	0.109 (18)	0.091 (16)	0.048 (11)	−0.020 (13)	0.006 (11)	−0.008 (10)
O33	0.17 (3)	0.11 (2)	0.11 (2)	0.08 (2)	−0.03 (2)	−0.008 (17)
O34	0.27 (5)	0.087 (19)	0.15 (3)	0.06 (2)	0.13 (3)	0.027 (19)

### Geometric parameters (Å, °)

**Table:**

Re1—C3	1.88 (3)	C23—C24	1.34 (5)
Re1—C2	1.93 (3)	C23—H23	0.9500
Re1—C1	1.98 (3)	C24—C25	1.34 (6)
Re1—O7	2.146 (14)	C24—H24	0.9500
Re1—N41	2.165 (17)	C25—C26	1.51 (4)
Re1—N31	2.22 (2)	C25—H25	0.9500
C1—O1	1.13 (3)	C26—H26	0.9500
C2—O2	1.15 (3)	N31—C32	1.32 (3)
C3—O3	1.20 (3)	N31—C36	1.33 (3)
Re2—C4	1.91 (2)	C32—C33	1.43 (3)
Re2—C6	1.93 (2)	C32—C42	1.46 (4)
Re2—C5	1.95 (3)	C33—C34	1.36 (4)
Re2—N11	2.171 (18)	C33—H33	0.9500
Re2—O7	2.185 (14)	C34—C35	1.38 (5)
Re2—N21	2.190 (18)	C34—H34	0.9500
C4—O4	1.15 (3)	C35—C36	1.43 (4)
C5—O5	1.17 (3)	C35—H35	0.9500
C6—O6	1.15 (3)	C36—H36	0.9500
O7—H7	0.8199	N41—C46	1.37 (3)
N11—C12	1.33 (4)	N41—C42	1.41 (3)
N11—C16	1.39 (4)	C42—C43	1.37 (4)
C11—C23	1.39 (4)	C43—C44	1.41 (4)
C11—N21	1.44 (3)	C43—H43	0.9500
C11—C12	1.47 (4)	C44—C45	1.41 (4)
C12—C13	1.40 (3)	C44—H44	0.9500
C13—C14	1.35 (5)	C45—C46	1.33 (4)
C13—H13	0.9500	C45—H45	0.9500
C14—C15	1.41 (4)	C46—H46	0.9500
C14—H14	0.9500	Re3—O34	1.56 (3)
C15—C16	1.40 (3)	Re3—O32	1.71 (2)
C15—H15	0.9500	Re3—O31	1.710 (18)
C16—H16	0.9500	Re3—O33	1.73 (3)
N21—C26	1.32 (3)		
			
C3—Re1—C2	89.7 (12)	C15—C16—H16	121.2
C3—Re1—C1	86.7 (9)	C26—N21—C11	122 (2)
C2—Re1—C1	88.0 (12)	C26—N21—Re2	123.2 (17)
C3—Re1—O7	92.6 (9)	C11—N21—Re2	114.8 (14)
C2—Re1—O7	172.7 (10)	C24—C23—C11	120 (3)
C1—Re1—O7	99.1 (8)	C24—C23—H23	120.2
C3—Re1—N41	174.9 (10)	C11—C23—H23	120.2
C2—Re1—N41	94.8 (9)	C23—C24—C25	125 (3)
C1—Re1—N41	95.9 (8)	C23—C24—H24	117.5
O7—Re1—N41	82.7 (6)	C25—C24—H24	117.5
C3—Re1—N31	102.1 (8)	C24—C25—C26	117 (3)
C2—Re1—N31	93.7 (11)	C24—C25—H25	121.7
C1—Re1—N31	171.1 (8)	C26—C25—H25	121.7
O7—Re1—N31	79.1 (6)	N21—C26—C25	119 (3)
N41—Re1—N31	75.2 (7)	N21—C26—H26	120.7
O1—C1—Re1	179 (2)	C25—C26—H26	120.7
O2—C2—Re1	174 (3)	C32—N31—C36	123 (2)
O3—C3—Re1	176.5 (18)	C32—N31—Re1	114.8 (17)
C4—Re2—C6	84.8 (9)	C36—N31—Re1	122.3 (18)
C4—Re2—C5	86.5 (11)	N31—C32—C33	119 (3)
C6—Re2—C5	89.6 (9)	N31—C32—C42	119 (2)
C4—Re2—N11	172.1 (9)	C33—C32—C42	122 (2)
C6—Re2—N11	91.2 (8)	C34—C33—C32	120 (3)
C5—Re2—N11	100.3 (9)	C34—C33—H33	120.0
C4—Re2—O7	99.7 (8)	C32—C33—H33	120.0
C6—Re2—O7	175.5 (7)	C33—C34—C35	120 (3)
C5—Re2—O7	90.3 (7)	C33—C34—H34	120.2
N11—Re2—O7	84.4 (6)	C35—C34—H34	120.2
C4—Re2—N21	98.5 (9)	C34—C35—C36	119 (3)
C6—Re2—N21	97.7 (7)	C34—C35—H35	120.6
C5—Re2—N21	171.5 (8)	C36—C35—H35	120.6
N11—Re2—N21	75.3 (7)	N31—C36—C35	120 (3)
O7—Re2—N21	82.2 (6)	N31—C36—H36	120.2
O4—C4—Re2	175 (2)	C35—C36—H36	120.2
O5—C5—Re2	176 (2)	C46—N41—C42	117.9 (19)
O6—C6—Re2	178 (2)	C46—N41—Re1	125.9 (15)
Re1—O7—Re2	132.8 (6)	C42—N41—Re1	115.6 (14)
Re1—O7—H7	113.6	C43—C42—N41	120 (2)
Re2—O7—H7	113.6	C43—C42—C32	126 (2)
C12—N11—C16	119 (2)	N41—C42—C32	113.5 (18)
C12—N11—Re2	118.4 (16)	C42—C43—C44	122 (3)
C16—N11—Re2	122.6 (18)	C42—C43—H43	119.2
C23—C11—N21	119 (3)	C44—C43—H43	119.2
C23—C11—C12	128 (3)	C45—C44—C43	115 (2)
N21—C11—C12	113.7 (19)	C45—C44—H44	122.4
N11—C12—C13	123 (3)	C43—C44—H44	122.4
N11—C12—C11	117 (2)	C46—C45—C44	123 (2)
C13—C12—C11	120 (3)	C46—C45—H45	118.7
C14—C13—C12	121 (4)	C44—C45—H45	118.7
C14—C13—H13	119.4	C45—C46—N41	122 (2)
C12—C13—H13	119.4	C45—C46—H46	118.8
C13—C14—C15	115 (3)	N41—C46—H46	118.8
C13—C14—H14	122.3	O34—Re3—O32	102.9 (18)
C15—C14—H14	122.3	O34—Re3—O31	104.5 (15)
C14—C15—C16	124 (3)	O32—Re3—O31	105.8 (13)
C14—C15—H15	118.1	O34—Re3—O33	130 (2)
C16—C15—H15	118.1	O32—Re3—O33	105.1 (15)
N11—C16—C15	118 (3)	O31—Re3—O33	106.2 (12)
N11—C16—H16	121.2		
			
C3—Re1—C1—O1	−168 (100)	C12—C11—N21—C26	176 (2)
C2—Re1—C1—O1	102 (100)	C23—C11—N21—Re2	169.9 (19)
O7—Re1—C1—O1	−76 (100)	C12—C11—N21—Re2	−11 (2)
N41—Re1—C1—O1	7(100)	C4—Re2—N21—C26	−4.7 (19)
N31—Re1—C1—O1	1(100)	C6—Re2—N21—C26	−90.6 (18)
C3—Re1—C2—O2	−19 (27)	C5—Re2—N21—C26	121 (5)
C1—Re1—C2—O2	68 (27)	N11—Re2—N21—C26	−179.8 (18)
O7—Re1—C2—O2	−127 (25)	O7—Re2—N21—C26	94.0 (18)
N41—Re1—C2—O2	164 (27)	C4—Re2—N21—C11	−178.0 (15)
N31—Re1—C2—O2	−121 (27)	C6—Re2—N21—C11	96.1 (15)
C2—Re1—C3—O3	102 (42)	C5—Re2—N21—C11	−53 (6)
C1—Re1—C3—O3	14 (42)	N11—Re2—N21—C11	6.9 (13)
O7—Re1—C3—O3	−85 (42)	O7—Re2—N21—C11	−79.3 (14)
N41—Re1—C3—O3	−106 (40)	N21—C11—C23—C24	3(4)
N31—Re1—C3—O3	−164 (41)	C12—C11—C23—C24	−176 (3)
C6—Re2—C4—O4	35 (28)	C11—C23—C24—C25	−1(5)
C5—Re2—C4—O4	125 (29)	C23—C24—C25—C26	−1(5)
N11—Re2—C4—O4	−25 (33)	C11—N21—C26—C25	1(3)
O7—Re2—C4—O4	−145 (28)	Re2—N21—C26—C25	−171.7 (18)
N21—Re2—C4—O4	−62 (29)	C24—C25—C26—N21	1(4)
C4—Re2—C5—O5	131 (33)	C3—Re1—N31—C32	170.4 (17)
C6—Re2—C5—O5	−144 (33)	C2—Re1—N31—C32	−99.1 (18)
N11—Re2—C5—O5	−53 (33)	C1—Re1—N31—C32	1(6)
O7—Re2—C5—O5	32 (33)	O7—Re1—N31—C32	80.1 (16)
N21—Re2—C5—O5	5(37)	N41—Re1—N31—C32	−5.1 (16)
C4—Re2—C6—O6	−85 (55)	C3—Re1—N31—C36	−11 (2)
C5—Re2—C6—O6	−171 (55)	C2—Re1—N31—C36	79 (2)
N11—Re2—C6—O6	88 (55)	C1—Re1—N31—C36	179 (5)
O7—Re2—C6—O6	101 (57)	O7—Re1—N31—C36	−102 (2)
N21—Re2—C6—O6	13 (56)	N41—Re1—N31—C36	173 (2)
C3—Re1—O7—Re2	108.3 (10)	C36—N31—C32—C33	8(4)
C2—Re1—O7—Re2	−143 (7)	Re1—N31—C32—C33	−174.1 (19)
C1—Re1—O7—Re2	21.2 (12)	C36—N31—C32—C42	−179 (2)
N41—Re1—O7—Re2	−73.6 (10)	Re1—N31—C32—C42	−1(3)
N31—Re1—O7—Re2	−149.9 (11)	N31—C32—C33—C34	−5(4)
C4—Re2—O7—Re1	13.1 (12)	C42—C32—C33—C34	−178 (3)
C6—Re2—O7—Re1	−173 (9)	C32—C33—C34—C35	−1(5)
C5—Re2—O7—Re1	99.5 (11)	C33—C34—C35—C36	6(5)
N11—Re2—O7—Re1	−160.1 (10)	C32—N31—C36—C35	−3(4)
N21—Re2—O7—Re1	−84.3 (10)	Re1—N31—C36—C35	179 (2)
C4—Re2—N11—C12	−40 (7)	C34—C35—C36—N31	−4(5)
C6—Re2—N11—C12	−99.6 (17)	C3—Re1—N41—C46	123 (8)
C5—Re2—N11—C12	170.6 (17)	C2—Re1—N41—C46	−86 (2)
O7—Re2—N11—C12	81.4 (16)	C1—Re1—N41—C46	2.2 (19)
N21—Re2—N11—C12	−2.0 (16)	O7—Re1—N41—C46	100.6 (18)
C4—Re2—N11—C16	136 (6)	N31—Re1—N41—C46	−178.8 (19)
C6—Re2—N11—C16	76 (2)	C3—Re1—N41—C42	−48 (9)
C5—Re2—N11—C16	−14 (2)	C2—Re1—N41—C42	103.1 (17)
O7—Re2—N11—C16	−102.9 (19)	C1—Re1—N41—C42	−168.5 (15)
N21—Re2—N11—C16	174 (2)	O7—Re1—N41—C42	−70.1 (14)
C16—N11—C12—C13	4(3)	N31—Re1—N41—C42	10.6 (14)
Re2—N11—C12—C13	−179.8 (18)	C46—N41—C42—C43	3(3)
C16—N11—C12—C11	−179 (2)	Re1—N41—C42—C43	174.5 (19)
Re2—N11—C12—C11	−3(3)	C46—N41—C42—C32	174.2 (19)
C23—C11—C12—N11	−171 (3)	Re1—N41—C42—C32	−14 (2)
N21—C11—C12—N11	9(3)	N31—C32—C42—C43	−179 (2)
C23—C11—C12—C13	5(4)	C33—C32—C42—C43	−7(4)
N21—C11—C12—C13	−174 (2)	N31—C32—C42—N41	10 (3)
N11—C12—C13—C14	−3(4)	C33—C32—C42—N41	−177 (2)
C11—C12—C13—C14	−180 (2)	N41—C42—C43—C44	−8(4)
C12—C13—C14—C15	−2(4)	C32—C42—C43—C44	−178 (2)
C13—C14—C15—C16	7(5)	C42—C43—C44—C45	9(4)
C12—N11—C16—C15	0(4)	C43—C44—C45—C46	−6(4)
Re2—N11—C16—C15	−176 (2)	C44—C45—C46—N41	2(4)
C14—C15—C16—N11	−6(5)	C42—N41—C46—C45	0(3)
C23—C11—N21—C26	−4(3)	Re1—N41—C46—C45	−170.4 (18)
